# Gender-affirming hormonal therapy induces a gender-concordant fecal metagenome transition in transgender individuals

**DOI:** 10.1186/s12916-024-03548-z

**Published:** 2024-09-02

**Authors:** Timur Liwinski, Matthias K. Auer, Johanna Schröder, Ina Pieknik, Christian Casar, Dorothee Schwinge, Lara Henze, Günter K. Stalla, Undine E. Lang, Alina von Klitzing, Peer Briken, Thomas Hildebrandt, Jeanne C. Desbuleux, Sarah V. Biedermann, Paul-Martin Holterhus, Corinna Bang, Christoph Schramm, Johannes Fuss

**Affiliations:** 1 Clinic for Adult Psychiatry, University Psychiatric Clinics, University of Basel, Wilhelm Klein-Strasse 27, Basel, CH-4002 Switzerland; 2 Medizinische Klinik and Poliklinik IV, Klinikum der Universität München, LMU München, Munich, Germany; 3 Institute of Forensic Psychiatry and Sex Research, Center for Translational Neuro- and Behavioral Sciences, University of Duisburg-Essen, Alfredstr. 68-72, Essen, 45130 Germany; 4 Department of Psychology, Institute for Clinical Psychology and Psychotherapy, Medical School Hamburg, Hamburg, Germany; 5 First Department of Medicine, University Medical Centre Hamburg-Eppendorf (UKE), Hamburg, Germany; 6 Medicover Neuroendocrinology, Munich, Germany; 7 Institute for Sex Research, Sexual Medicine and Forensic Psychiatry, University Medical Center Hamburg-Eppendorf, Hamburg, Germany; 8 Department of Gynecology and Obstetrics, CCC Erlangen EMN, Friedrich Alexander University, Erlangen, Germany; 9 Department of Psychiatry and Psychotherapy, Social and Emotional Neuroscience Group, Center for Psychosocial Medicine, University Medical Center Hamburg-Eppendorf, Hamburg, Germany; 10 Division of Pediatric Endocrinology and Diabetes, Department of Children and Adolescent Medicine I, University Hospital of Schleswig-Holstein, Campus Kiel/Christian-Albrechts University of Kiel, Kiel, D-24105 Germany; 11 Institute of Clinical Molecular Biology, Christian-Albrechts-University Kiel, University Hospital Schleswig-Holstein, Rosalind-Franklin-Str. 12, Kiel, 24105 Germany; 12 Hamburg Centre for Translational Immunology (HCTI), University Medical Centre Hamburg-Eppendorf, Hamburg, Germany; 13 Martin Zeitz Center for Rare Diseases, University Medical Center Hamburg-Eppendorf, Hamburg, Germany

**Keywords:** Gender-affirming hormonal therapy, Sex steroids, Microbiome, Microbiota, Metagenome, Trans women, Trans men, Fatty acid-related metabolism

## Abstract

**Background:**

Limited data exists regarding gender-specific microbial alterations during gender-affirming hormonal therapy (GAHT) in transgender individuals. This study aimed to investigate the nuanced impact of sex steroids on gut microbiota taxonomy and function, addressing this gap. We prospectively analyzed gut metagenome changes associated with 12 weeks of GAHT in trans women and trans men, examining both taxonomic and functional shifts.

**Methods:**

Thirty-six transgender individuals (17 trans women, 19 trans men) provided pre- and post-GAHT stool samples. Shotgun metagenomic sequencing was used to assess the changes in gut microbiota structure and potential function following GAHT.

**Results:**

While alpha and beta diversity remained unchanged during transition, specific species, including *Parabacteroides goldsteinii* and *Escherichia coli*, exhibited significant abundance shifts aligned with affirmed gender. Overall functional metagenome analysis showed a statistically significant effect of gender and transition (*R*^2^ = 4.1%, *P* = 0.0115), emphasizing transitions aligned with affirmed gender, particularly in fatty acid-related metabolism.

**Conclusions:**

This study provides compelling evidence of distinct taxonomic and functional profiles in the gut microbiota between trans men and women. GAHT induces androgenization in trans men and feminization in trans women, potentially impacting physiological and health-related outcomes.

**Trial registration:**

Clinicaltrials.gov NCT02185274.

**Supplementary Information:**

The online version contains supplementary material available at 10.1186/s12916-024-03548-z.

## Background

Transgender individuals experience a mismatch between their sex assigned at birth and their gender identity [1]. Historically, transgender identities were seen as rare, but recent data indicate that this population is growing. Current estimates suggest that approximately 0.3 to 0.5% of adults and 1.2 to 2.7% of children and adolescents identify as transgender [2]. When considering broader gender diversity, these numbers increase from 0.5 to 4.5% among adults and 2.5 to 8.4% among younger populations [2]. In the USA, about 1.6 million individuals aged 13 and older identified as transgender in 2022 [3], with a recent Pew survey indicating that 5% of adults under 30 identify as transgender or gender non-binary [4]. Transgender individuals frequently face stigma, discrimination, and social exclusion, leading to various health disparities [5, 6]. Seminal studies, such as the Trans PULSE Project, highlight how anti-trans stigma has led to the erasure of transgender individuals in health research, policy, and practice [7]. Despite recent increases in global transgender health research, significant gaps remain, particularly in high-quality studies [8, 9]. One notable gap is research on gender-specific microbial alterations during gender-affirming hormonal therapy (GAHT), commonly referred to as hormone replacement therapy (HRT). A large percentage of transgender individuals either use or plan to use GAHT to develop physical traits aligned with their desired gender, thereby affirming their gender identity and enhancing their mental health and quality of life. In the 2015 U.S. Transgender Survey, 78% of respondents desired hormone therapy related to gender transition, but only 49% had ever received it [10]. Gender affirmation is a personalized process and has been associated with positive health outcomes, while attempts to alter a person’s gender identity, such as so-called reparative or conversion therapy, cause substantial harm [11–14].

The human gut microbiota, consisting of trillions of microorganisms in the gastrointestinal tract, plays a crucial role in overall health and interacts with almost every organ system [15]. Research into the relationship between sex-specific microbiota and clinical characteristics is still emerging. Some studies suggest differences in microbiota composition between genders, but results have been inconsistent [16–20]. The relationship between sex hormones and gut microbiota is complex and bidirectional: specific bacteria can influence sex steroid metabolism, while sex steroids may affect microbiota composition [18, 21–26]. Exploring these interactions in humans is challenging due to numerous confounding factors, such as genetic background, which significantly impacts microbiota composition [27]. Cross-sectional microbiota studies cannot establish causative relationships, making prospective research essential [28]. Transgender individuals undergoing GAHT provide a unique opportunity to study sex steroids’ effects on gut microbiota, as their chromosomal background remains stable while they undergo significant hormonal changes. Investigating the interplay between gender, sex hormones, and microbiota addresses a critical gap in medical knowledge. Understanding these interactions is vital for developing improved diagnostic and therapeutic strategies for conditions influenced by hormonal status and gut microbiota, such as autoimmune diseases, colorectal cancer, metabolic syndrome, and mental health disorders [29–32]. Furthermore, research on GAHT’s impact on gut microbiota could directly benefit transgender individuals. The microbiome, often termed the “second genome,” is crucial in health and disease, correlating with various health markers and offering potential therapeutic targets [33–36]. As microbiome research progresses, its integration into clinical practice is increasing, promising new insights and interventions [37]. However, transgender individuals remain underrepresented in microbiota studies, hindering our understanding of how microbiota changes affect their health [38]. They face higher risks for various health conditions, including cardiovascular disease, HIV-related illnesses, cancers, and suicide [39]. Therefore, a multidisciplinary approach is necessary for effective transgender healthcare, and primary care practitioners play a crucial role [14, 40]. With the growing transgender population, there is a pressing need for healthcare providers to receive education on the complexities of transgender healthcare to ensure equitable care.

This study presents the results of a prospective investigation into the impact of 12 weeks of GAHT on the gut metagenome, using metagenomic shotgun sequencing. It aims to fill the existing knowledge gap by providing a comprehensive analysis of how GAHT affects gut microbiota in trans women and trans men. The findings will have implications for clinical care and future research, aiming to inform primary care practitioners and specialists involved in transgender healthcare.

## Methods

### Sex as a biological variable

This study, involving transgender individuals undergoing gender-affirming hormonal therapy (GAHT), was meticulously designed to consider sex as a critical biological variable. Given the unique nature of the study population, which includes both trans women and trans men, the focus inherently involves exploring the impact of GAHT on individuals assigned female at birth and individuals assigned male at birth. This approach aligns with the aim of understanding the nuanced effects of sex steroids on gut microbiota in the context of gender transition. This design choice recognizes the importance of capturing the diversity of responses based on sex and ensures a comprehensive exploration of microbial alterations associated with GAHT. As such, the findings are anticipated to contribute insights applicable to individuals across the biological sex spectrum, enriching our understanding of the interplay between sex hormones and the gut microbiota in the transgender population.

### Study design

The data analyzed here were collected within the “Transgender in Transition” (acronym: Transit) study, a prospective multicenter observational study aiming at the assessment of the effects of GAHT on psychological and metabolic endpoints. In this research article, we focused on analyzing, evaluating, and interpreting a specific aspect of the collected materials, namely the stool samples. Stool samples were obtained from transgender individuals before and 3 months after initiation of GAHT. Serum hormone levels were measured to confirm effectiveness and compliance with therapy. Food intake was assessed using a self-constructed item questionnaire obtaining information on all relevant consumed nutrients at each time point (Additional File 1). We analyzed potential variations in food intake frequency in order to more precisely delineate the influence of sex steroids on gut microbiota from potential alterations in dietary patterns that may be associated with the transitional phase.

The present data were collected between June 2014 and September 2021 in four different centers, including the Endocrinology Department of the Max Planck Institute of Psychiatry in collaboration with the Hormone and Metabolism Center Munich, the Gynecology Department of the University Hospital Erlangen, and the Institute for Sex Research, Sexual Medicine, and Forensic Psychiatry of the University Medical Center Hamburg-Eppendorf.

### Study population

The recruitment of participants included in the study was conducted by the scientific staff and physicians from the medical departments.

- Inclusion criteria: transsexualism according to ICD-10 (F64.0)
- Exclusion criteria: age < 18 years, incapacity for legal transactions due to other reasons, severe medical comorbidity, pregnancy, and use of antibiotics within 4 weeks before stool collection

Based on these criteria, we were able to include samples from *n* = 36 participants in the study, including 17 trans women and 19 trans men. No non-binary individuals were included in the study because our center did not receive any requests for gender trait-modulating hormonal therapy from non-binary individuals.

Some individuals received psychiatric and/or somatic diagnoses, which were evenly distributed. Among transgender men, depression (F32.9) was the most common diagnosis with four cases, followed by Hashimoto thyroiditis (E06.3) with two cases. Additionally, there was one case each of autism (F84.0), asthma (J45.0), ulcerative colitis (K51.9), coronary artery disease (I25.10), irritable bowel syndrome (K58.9), and gastroesophageal reflux disease (K21.9). For transgender women, depression (F32.9) was diagnosed in two cases, while cholesteatoma (H71.90) and alopecia universalis (L63.0) each had one case. Anxiety disorder (F41.9) was also diagnosed in one case among transgender women.

The initial assessment was conducted before the start of GAHT. Clinical data was collected prospectively. Twelve trans women and 13 trans men returned a completed food item frequency questionnaire before and 3 months after the commencement of GAHT. The collection of blood samples, as well as measurements of body mass index (BMI), were carried out in the medical centers. Participants were given stool collection tubes with DNA stabilizer (Invitek Diagnostics, Berlin, Germany). Follow-up assessments were scheduled 3 months after the commencement of GAHT. Participants with antibiotic intake within the 3-month study period were not included in the analysis.

All participants in the study received standard full doses of sex steroids. Hormone therapy for trans men was administered by the responsible endocrinologists and consisted of either transdermal testosterone gel (25–50 mg testosterone per day), injections of 1000 mg testosterone undecanoate every 12–14 weeks, or testosterone enanthate 250 mg every 2–3 weeks. The target testosterone levels just before the next injection were within the mid-range of age-adjusted reference values [41]. Trans women were treated with either estradiol transdermal gel (1.2–3.6 mg 17b-estradiol per day) or oral estradiol valerate (2–8mg per day) and oral cyproterone acetate (5–50 mg per day). The goal was to achieve normal or suppressed luteinizing/follicle-stimulating hormone levels (since participants had not undergone gonadectomy) with estradiol levels in the mid-follicular range approximately 2 to 4 h after application, as well as testosterone levels within the female reference range [41].

### Hormonal measurements

Serum was obtained by centrifugation at 2000*g* for 10 min and frozen at − 80°C until further analysis. Quantitative measurement of serum hormone levels was performed using MassChrom Steroid LC–MS/MS Assays (Chromsystems, Germany) by UPLC-ESI–MS mass spectrometry LCMS-8060 (Shimadzu, Japan). The steroid measurements were carried out at the UKSH in the pediatric endocrinology laboratory at the Department of Pediatrics and Adolescent Medicine I (Kiel, Germany). The laboratory is accredited according to DIN-ISO 15189.

### Stool sample material

After stool sample collection using stool collection tubes with DNA stabilizer (Invitek Diagnostics, Berlin, Germany; for kit instructions see Additional File 2) in the participants’ homes, the samples were immediately shipped by mail. The samples, taken at room temperature, were cooled to − 80°C upon arrival at the collaborating microbiota laboratory in Kiel and stored until further processing.

### DNA extraction

DNA extraction was performed using the QIAamp Fast DNA Stool Mini Kit (Qiagen, Hilden, Germany) on the QIAcube automated system. Approximately 200 mg of stool sample was transferred to 0.70-mm Garnet Bead tubes, which were filled with 1.1 ml of ASL lysis buffer (containing proteinase K). Subsequently, the cells were lysed using bead-beating as the cell disruption method, where cell suspensions in reaction vessels are disrupted by small glass beads through mechanical effects. The homogenizer SpeedMill PLUS was used for 45 s at 50 Hz for this purpose. Afterward, the samples were heated at 95°C for 5 min. The purification of the sample was ensured by the QIAamp silica membrane, which allows for the passage of impurities while specifically binding the DNA. PCR inhibitors were removed using an optimized buffer. The amount of extracted DNA ranged from 1 to 10 µg per sample.

### Shotgun metagenomic analysis

Shotgun metagenomic analysis was conducted on the samples. Instead of focusing solely on individual genes like those responsible for ribosomal RNA (rRNA) to generate taxonomy-based “16S rRNA profiles,” shotgun metagenomic sequencing encompasses the sequencing of the entire metagenome of microbial communities. In contrast to 16S sequencing, which offers limited taxonomic information, shotgun metagenomic sequencing (MGS) of the stool microbiome provides comprehensive insights into both taxonomic composition and functional attributes [37]. This approach has demonstrated significant promise in terms of diagnostic utility [37].

The Illumina DNA Prep Library Preparation kit protocol was followed for sample preparation, and sequencing was performed on the NovaSeq Platform using 2 × 150 bp paired-end reads. The fastq files were generated using Illumina’s bcl2fastq script. To ensure data quality, reads were trimmed using Trimmomatic. To remove host reads, bowtie2 (v2.3.5.1) was employed against the GRCh38 human reference genome. Taxonomic assignment of bacterial DNA was performed using Kraken2 (v2.1.3) against the Genome Taxonomy Database, relying on the exact alignment of k-mers. Bayesian re-estimation of bacterial abundance was carried out using Bracken (v2.8). Bacterial taxa with a total abundance of less than 0.001% were excluded. For functional annotation, the Humann3 pipeline was utilized, using the same input reads as for the taxonomic analysis. The output tables were normalized to relative abundances, combined, and annotated to gene families based on UniRef protein clusters (90% identity), as well as to the Kyoto Encyclopedia of Genes and Genomes (KEGG) and Gene Ontology (GO) terms.

### Statistics

All statistical analyses were performed using the R software (v3.5.1). Normality was assessed employing the Shapiro test. In instances of normally distributed data among independent samples, the Welch *t*-test was employed, while non-normally distributed data was subjected to the Wilcoxon test. The examination of food frequency data involved a repeated-measures two-way analysis of variance (ANOVA) utilizing the R stats aov function, incorporating an interaction term for time and type of transition.

To assess within-sample diversity (α-diversity), we calculated Shannon entropy based on a Hellinger-transformed abundance matrix using the “vegan” package (v2.5.6) functions “decostand” and “diversity” [42]. We employed the *F*-test to compare variances of the Shannon index. Additionally, we employed a generalized additive mixed model to test for the statistical significance of differences in alpha diversity, utilizing the “mgcv” R package, version 1.9–0 [43]. This approach was chosen to account for repeated measurements and random effects arising from multiple assessments of the same patient. The regression estimate (*β*) ± and the standard error (SE) are reported.

For evaluating between-sample diversity (*β*-diversity), we used Bray–Curtis dissimilarity on Hellinger-transformed species abundance and metabolic pathway relative abundance matrices. The statistical significance of separation among groups was assessed using permutational multivariate analyses of variance (PERMANOVA). Pairwise multilevel comparisons with PERMANOVA, while considering repeated measures, were conducted employing the 'pairwiseAdonis' function (https://github.com/pmartinezarbizu/pairwiseAdonis).

Differences in individual bacterial species were tested using the generalized additive mixed model (GAMM; “mgcv” package, version 1.9–0) on the untransformed count data matrix, with library size as an offset, assuming a negative binomial distribution. The same models were employed to test for differences in the distribution of single pathways, assuming a Gaussian distribution.

To assess the predictive capability of the previously identified sex-associated species, predictive modeling was performed using a generalized linear model with the “rms” package (version 6.7–1), and the c-statistic (area under the receiver operating characteristic curve) was calculated to evaluate model performance.

For predicting clinical phenotypes based on single species-level and pathway-level abundances, we employed a method known as “selbal” [44]. This method operates with microbial balances, which are ratios of taxonomic quantities, allowing for feature selection and classification or regression while accounting for the compositional nature of microbial sequencing data and controlling for confounders. We used threefold cross-validation to obtain reliable results for each selbal model, and the area under the receiver operating characteristic curve (AUROC) was reported to assess classification performance.

The within-subject design enabled participants to serve as their own controls, thereby controlling for a range of known confounders that could potentially impact gut microbiota composition, such as age, metabolic status, lifestyle factors, comorbidities, medications, and diet. Additionally, we assessed dietary habits to ensure they remained consistent throughout the observation period.

*P*-values were generally considered significant when *P* < 0.05 and were adjusted for the false discovery rate (FDR) when necessary.

### Study approval

The study was approved by local ethics committees and is additionally registered in the US clinical trials registry (clinicaltrials.gov NCT02185274). It adheres to the ethical guidelines of the Helsinki Declaration of 1964 and its latest revision in 2013, as well as the STROBE Statement [45]. All participants were provided with information about the voluntary nature of participation, and written consent was obtained from all included subjects.

## Results

### Demographic, clinical, and dietary profile

In total, high-quality metagenomic samples from both time points, baseline (BL) and 3 months after transition initiation (M3) were available for *n* = 36 participants. Overall, 19 individuals underwent the transition from female to male hormonal phenotype (trans men) and 17 participants transitioned from male to female hormonal phenotype (trans women) (Table 1). As anticipated, there were alterations in sex hormone levels observed from baseline to 3 months following GAHT (Additional File 3: Fig. S1A-F). No significant adverse effects were reported, such as vomiting, constipation, diarrhea, significant weight loss or gain, thrombosis, serious mood changes, recurrent hot flashes, or severe headaches. Analyzing the food frequency questionnaire data, no discernible changes were observed in any food item concerning transition time point and transition type (all FDR > 0.1). This suggests that potential alterations in the microbiota are not attributable to significant dietary shifts during the transition period.

**Table 1:** Demographic, clinical, and behavioral characteristics of the study participants included in the microbiota analysis

Variable	Variable category	Transgender women (MTF; *n* = 17)	Transgender men (FTM; *n* = 19)	*P*-value
Age, years (mean, SD)		48.0 ± 17.2	24.4 ± 8.9	0.034
Body mass index, kg/m^2^ (mean, SD)		22.7 ± 3.5	23.7 ± 4.0	0.927
Hormone therapy (*n*, %)	Cyproterone acetate	12 (70.6)	NA	
	Estrogen/estradiol	17 (100)	NA	
	Finasteride	2 (11.8)	NA	
	Testosterone gel (transdermal)	NA	6 (31.6)	
	Testosterone undecanoate	NA	12 (63.2)	
	Testosterone enanthate	NA	1 (5.3)	

### Changes in gut microbial diversity in response to gender-affirming therapy

**Fig. 1:**
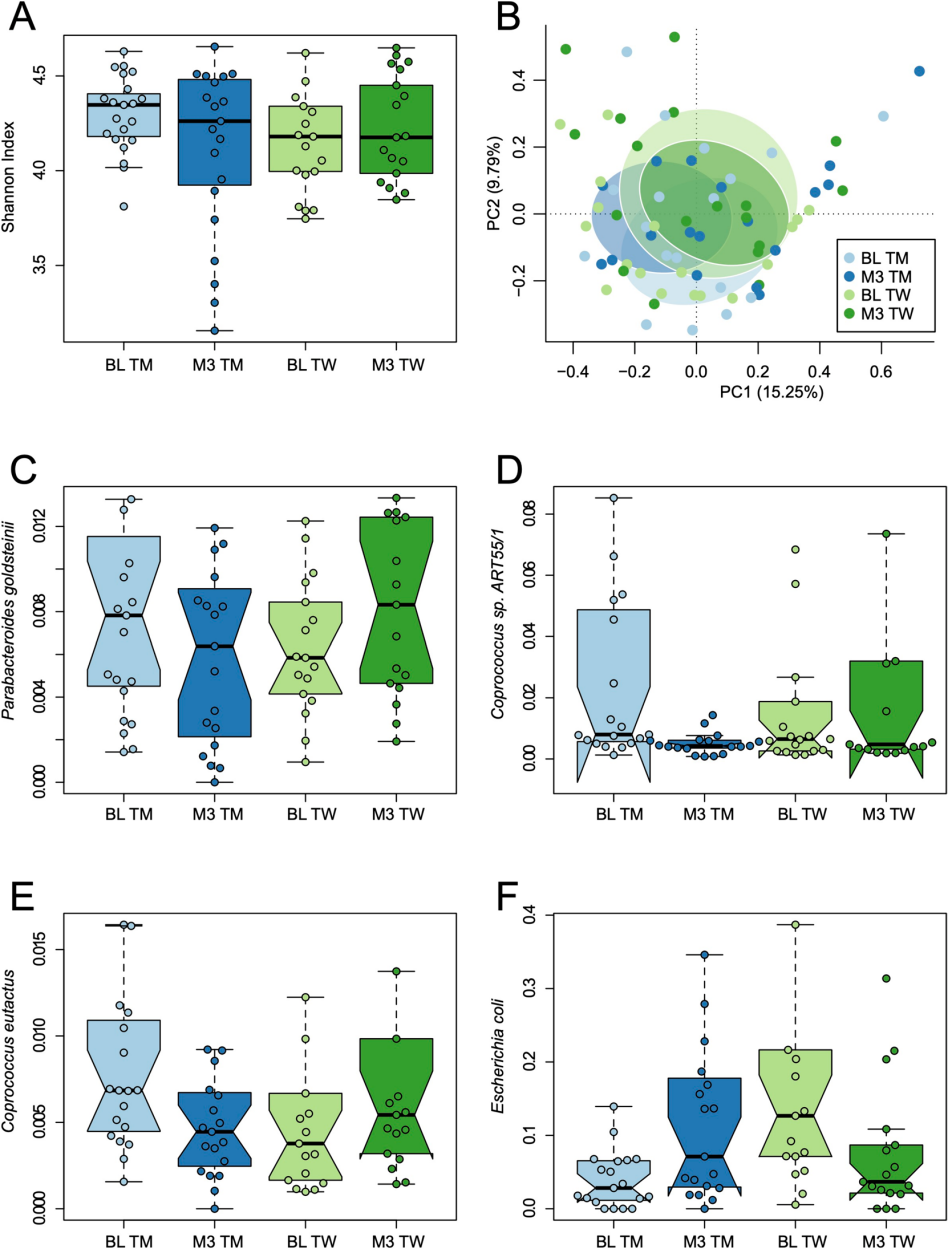
Changes in fecal microbiota composition in people with gender-affirming hormonal care. The figure shows the alpha (**A**) and beta (**B**) diversity, alongside the relative abundance distribution (Hellinger transformed) of species that exhibit statistically significant differential abundance (FDR < 0.05; **C–F**) between baseline (BL) and 3 months of gender-affirming hormonal care (M3). BL baseline, M3 3 months after commencement of gender-affirming hormonal care, TM trans men, TW trans women

We employed a GAMM to evaluate the effects of various factors, including transition type, time point, and their interaction, on alpha diversity (within-sample diversity), as measured by the Shannon index. Our analysis did not find significant differences in alpha diversity when considering the factors of transition type (β =  − 0.12 ± 0.10, *P* = 0.2672), time point (β =  − 0.16 ± 0.09, *P* = 0.0918), or their interaction (β = 0.19 ± 0.14, *P* = 0.1628; Fig. 1A). We assessed alterations in the variance of alpha diversity over time within two distinct groups: trans men and trans women. Our findings revealed a significant increase in the variance of the Shannon index within trans men (*F* = 0.22, *P* = 0.0024). c 3 months following the initiation of hormonal treatment (*F* = 1.22, *P* = 0.6941; Fig. 1A). This disparity in variance dynamics suggests differential responses to GAHT in these two distinct populations with regard to the alpha diversity of their gut microbiota.

We proceeded to examine the potential response of beta diversity (between-sample diversity) to GAHT. Following confirmation that the assumption of multivariate homogeneity of group dispersions (variances) was not violated (betadisper, *F* = 1.04, *P* = 0.3788), we conducted pairwise multilevel comparisons with PERMANOVA while considering repeated measures. Our analysis did not reveal a discernible overall effect of time (*R*^2^ = 2.16%, *P* = 0.0622), transition type (*R*^2^ = 1.1%, *P* = 0.0602), or their interaction (*R*^2^ = 1.7%, *P* = 0.1979) on microbiota composition (Fig. 1B). These results indicate that, within the scope of this study, there were no significant alterations in taxonomic beta diversity associated with GAHT over time or in relation to different transition types.

### Sex-dependent variations in bacterial species abundance following hormonal treatment

We proceeded with the analysis of changes in the relative abundance of individual bacterial species over the course of 3 months of GAHT. Following the application of the FDR, our investigation revealed four bacterial species that exhibited varying relative abundance patterns in relation to both the type of transition and time (Fig. 1C–F). These four species are as follows: *Parabacteroides goldsteinii* (β = 2.25 ± 0.64, FDR = 0.0264), *Coprococcus sp. ART55/1* (β = 3.75 ± 0.78, FDR = 3 × 10^–4^), *Coprococcus eutactus* (β = 2.16 ± 0.63, FDR = 0.0289), and *Escherichia coli* (β =  − 4.2 ± 0.99, FDR = 0.0019). Remarkably, in the case of these four bacterial species, we noted a distinctive pattern that aligns with the current sex steroid phenotype (pre- and post-transition) of the individuals. For instance, *Escherichia coli* exhibit on average a higher relative abundance in both trans women before hormonal therapy and trans men after hormonal therapy (i.e., a male-typical sex steroid phenotype), while displaying lower abundance in pre-transition trans men and post-transition trans women (i.e., a female-typical sex steroid milieu). Conversely, the *Coprococcus* species demonstrated a higher abundance in participants with a female-typical sex hormonal phenotype and a lower abundance in a male-typical hormonal phenotype, reflecting a notable association with hormonal sex state.

### Bacterial abundance predicts hormonal sex

**Fig. 2:**
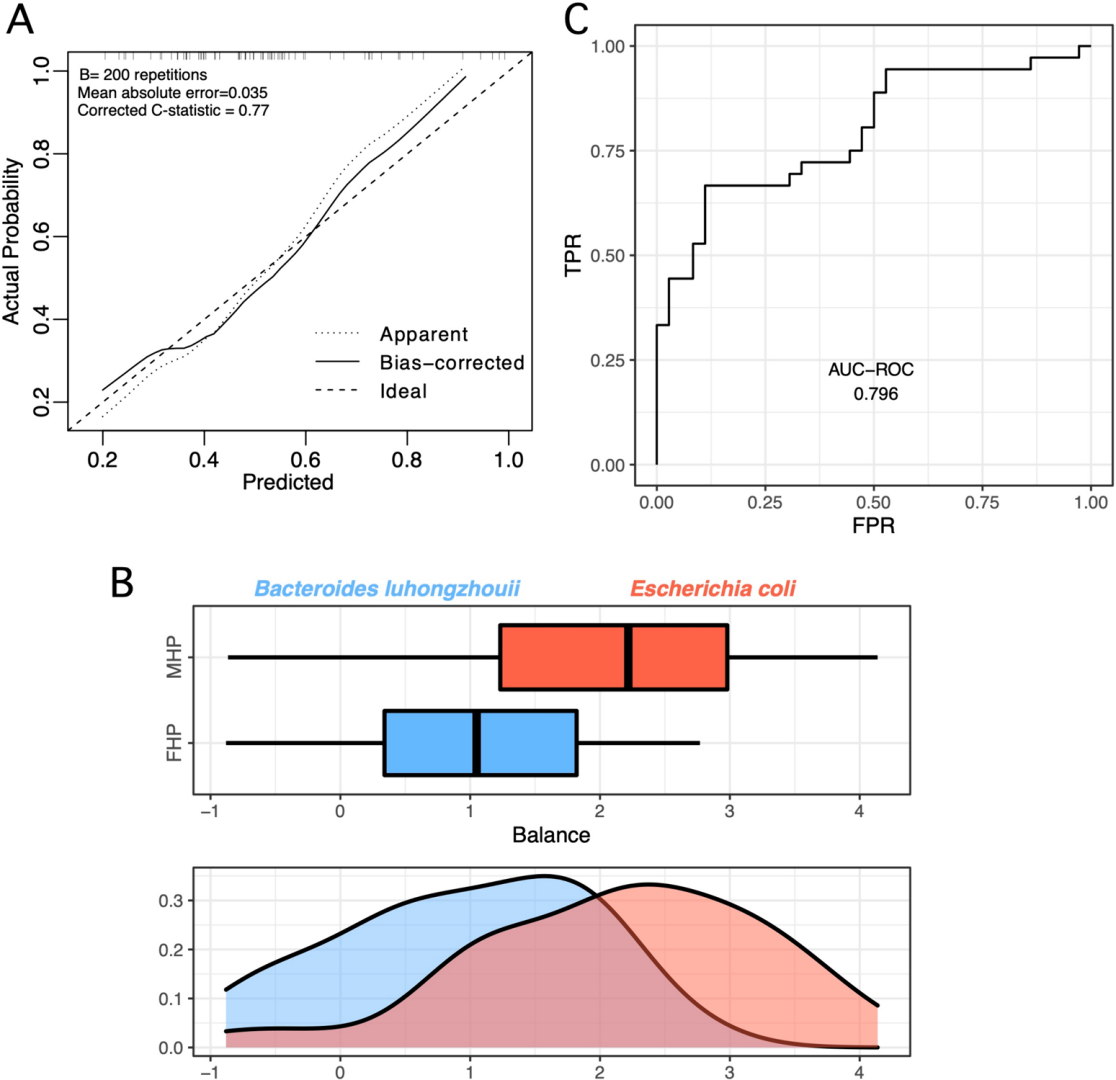
Ability of fecal microbiota composition to predict sex hormone phenotype. **A** The outcomes of predictive modeling employing a generalized linear model for distinguishing between the male-typical (MHP) and the female-typical sex hormone phenotype (FHP). The calibration curve demonstrates accurate predictions throughout the spectrum of predicted values. **B** Presentation of the global balance concerning the hormonal sex phenotype. The box plot illustrates the distribution of balance scores for female and male-typical hormonal phenotypes, specifying the two taxa groups constituting the global balance along with their respective density curves. **C** Depiction of the ROC curve accompanied by its corresponding AUC value (0.796). FHP male-typical hormonal phenotype, MHP male-typical hormonal phenotype

In order to evaluate the predictive capability of the previously identified sex steroid-associated species, we conducted predictive modeling utilizing a generalized linear model. In this model, we treated current hormonal sex phenotype as a binary variable and employed the four species as predictor variables. Specifically, we compared pre- and post-transition female with pre- and post-transition male-typical hormonal phenotypes. Our analysis involved the following steps: initially, we fitted the full model, and subsequently, we conducted a grid search to determine the optimal penalty value. To obtain an out-of-sample estimate of model performance, we employed bootstrap resampling. The resultant c-statistic, with a value of 0.77, indicates a good discriminatory ability of the model. Furthermore, the calibration curve demonstrates that the predictions closely align with the ideal predictions across the entire spectrum of predicted values (Fig. 2A).

We adopted an alternative predictive methodology that involved the inclusion of all accessible bacterial species, following an initial filtration step. In this approach, we employed the Selbal algorithm, which applies a forward-selection technique to identify two distinct groups of taxa. The focus was on taxa whose relative balance is associated with the response variable of interest. The Selbal analysis pinpointed a noteworthy shift in balances, which are essentially ratios of genus relative abundances. Specifically, the shift was observed between *Escherichia coli*, representing the numerator and indicative of male-typical sex hormone phenotype, and *Bacteroides luhongzhouii*, serving as the denominator and indicative of female-typical sex hormone phenotype (Fig. 2B). The observed shift in balance effectively discriminates between female and male individuals, achieving an area under the receiver operating characteristic curve (AUROC) of 79.6% (Fig. 2C).

### Alterations in microbial functionality in response to gender-affirming hormonal treatment

**Fig. 3:**
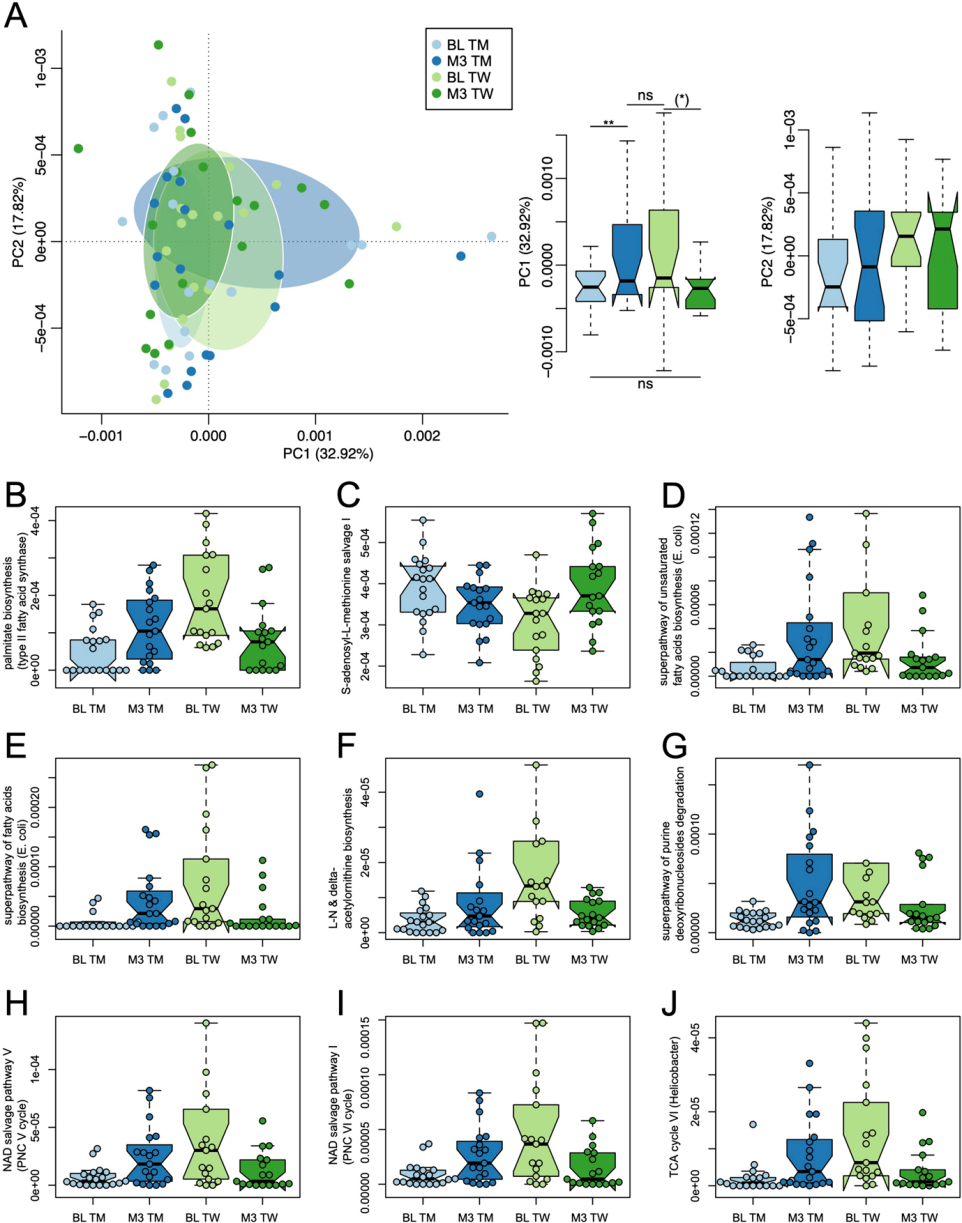
Metabolic pathway analysis. **A** The biplot visually represents the metagenomic functional beta-diversity; the graph shows a clear separation along the first principal coordinate (PC1), which captured most of the variation (32.92%). **B–J** Display of individual metagenomic pathways with statistically significant differential abundance at an FDR level < 0.001. BL baseline, M3 3 months after commencement of gender-affirming hormonal care, TM trans men, TW trans women, **p < 0.01; *p < 0.1; ns not significant; significance levels are indicated for post hoc paired *t*-tests

We conducted an in-depth analysis of the comprehensive functional metabolic capacity by examining the entire bacterial metagenome through the HUMAnN framework. Our analytical approach involved performing pairwise multilevel comparisons utilizing PERMANOVA while accounting for repeated measures. In the course of our investigation, we identified noteworthy associations in terms of overall functional metagenomic variation. Specifically, we observed that the sex assigned at birth (*R*^2^ = 1.7%, *P* = 0.0398) displayed significant associations with the functional metagenomic profile. Furthermore, our analysis revealed a statistically significant effect (*R*^2^ = 4.1%, *P* = 0.0115; Fig. 3A) associated with the interaction between transition type and time on the overall functional metagenome. Principal coordinate 1 (PC1), which captured most of the variation (32.92%), revealed a separation pattern that aligns with the current sex steroid phenotype (pre- and post-transition) of the individuals (*F* = 8.0, *p* = 0.0078; Fig. 3A). This finding underscores the influence of GAHT in inducing changes within the gut metagenome that align with the desired characteristics of the affirmed gender.

In order to pinpoint the pathways that exhibit alterations corresponding to hormonal sex status across the transition period, we employed the GAMM framework. Out of the 500 pathways identified by HUMAnN 3.0, a substantial portion, namely 184 pathways (representing 36.8% of the total), displayed differential abundance patterns. Following the application of FDR correction, our analysis unveiled that 125 pathways (25% of all pathways) exhibited a significant association with the process of transition (Additional File 4). This underscores the substantial extent of functional changes that are linked to hormonal sex status. Within this set, nine pathways demonstrated particularly robust associations with transition, as indicated by FDR values < 0.001 (Fig. 3B–J). Notably, akin to the single species analysis conducted earlier, all of the most prominent pathway hits’ average abundance values align with the characteristics of the target sex phenotype, further emphasizing the significant influence of hormonal sex milieu on the functional metabolic capacity of the microbiota.

**Fig. 4:**
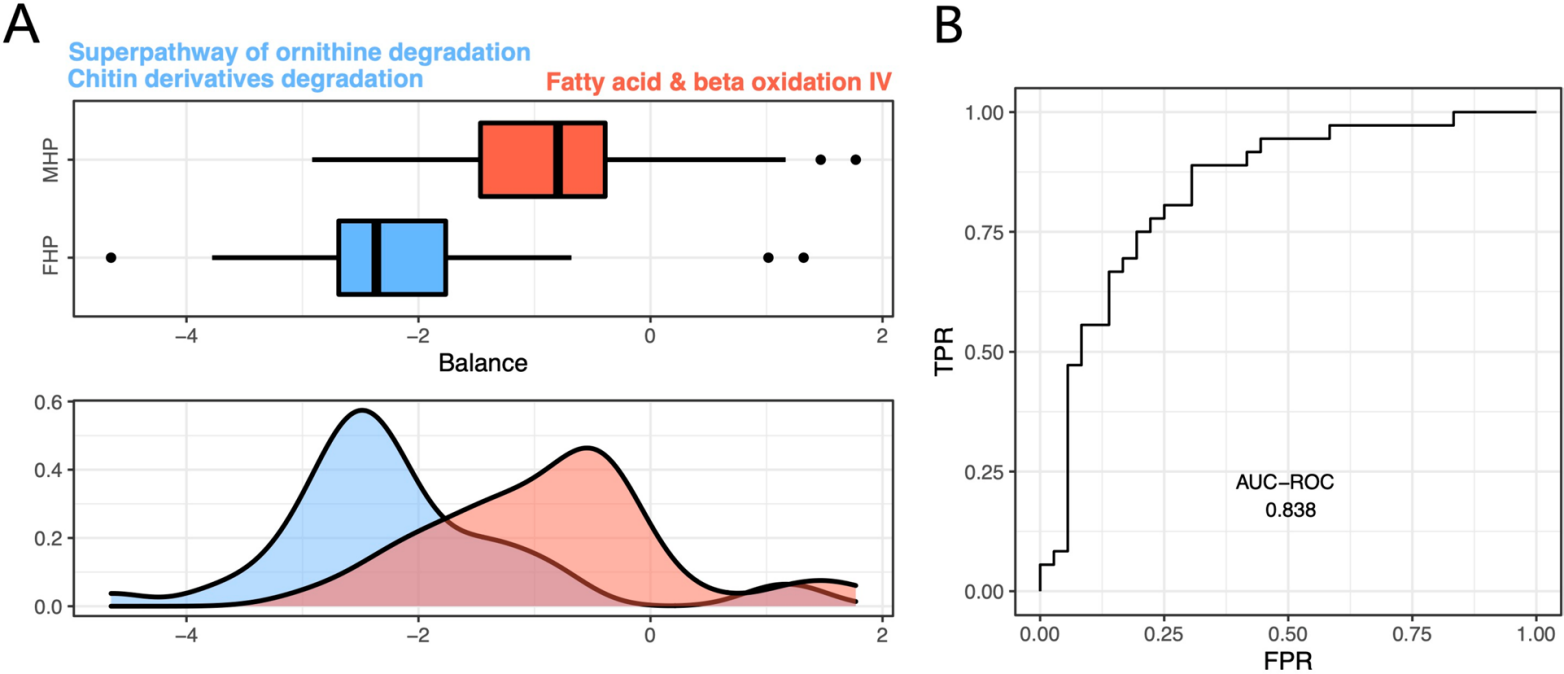
**A** Discriminatory capability of metabolic pathways for sex hormone status. Visualization of the global balance pertaining to the metagenomic functional hormonal sex phenotype. The box plot delineates the distribution of balance scores for female-typical (FHP) and male-typical hormonal phenotypes (MHP), identifying the two pathway groups composing the global balance, and providing their respective density curves. **B** ROC curve with its associated AUC value (0.838). FHP male-typical hormonal phenotype, MHP male-typical hormonal phenotype

In our attempt to further refine and focus on potentially pivotal alterations in pathways, we employed the Selbal methodology, which specifically examines the concept of balances within pathway data. This analysis led to the identification of an optimal discriminatory set consisting of three pathways, effectively distinguishing between individuals of both female- and male-typical hormonal phenotype (comprising both pre and post-transitional sexual hormonal statuses). Notably, among these pathways, “chitin derivatives degradation” and the “superpathway of ornithine degradation” emerged as characteristic features associated with the female group, while “fatty acid and beta oxidation IV” was identified as the hallmark of the male group. The balance observed among these three pathways exhibited a considerable discriminatory capacity, yielding an impressive area under the curve (AUC) of 83.8% (Fig. 4).

## Discussion

This study reveals significant gender-concordant changes in the taxonomic composition and metabolic capacity of fecal microbiota in a cohort of trans women and trans men undergoing GAHT. Previously, there was some evidence that sex might exert an influence on the diversity, composition, and function of the gut bacterial microbiota, although the findings have been inconclusive. Early investigations, involving a relatively small sample size and constrained by limited technological capabilities, yielded minimal to no discernible gender-related distinctions. For instance, a 2005 study involving 91 individuals of northern European descent (from France, Denmark, Germany, the Netherlands, and the UK) did not reveal any significant variations in gut microbiota between the sexes, as determined by principal component analysis [46]. In a 2008 Chinese study employing group-specific denaturing gradient gel electrophoresis (DGGE) profiling of *Bacteroides* species, a greater prevalence of *Bacteroides thetaiotaomicron* was observed in men [47]. Recent extensive population-wide studies have similarly not demonstrated significant sex-specific variations in the diversity, intricacy, or composition of gut microbiota [16, 48]. Nevertheless, these initial studies were methodologically limited, mainly due to the absence of shotgun metagenomic sequencing in most of them. In a substantial cohort study encompassing two independently well-characterized groups, namely the Belgian Flemish Gut Flora Project (consisting of 1106 individuals) and the Dutch LifeLines-DEEP study (comprising 1135 individuals), sex exhibited a relatively minor effect size among the 69 factors that were found to be significantly associated with the overall variation in the microbiota [49]. In a large Dutch research study employing metagenomic shotgun sequencing and after accounting for multiple variables, the only discernible association with sex was related to *Akkermansia muciniphila*, where women were observed to have a higher prevalence of this particular species [50]. Nevertheless, the outcomes of each study pertaining to variations in microbial taxa between the sexes display inconsistencies [50–53]. This lack of consistency is to be expected, given the myriad potential confounding variables and the multitude of factors related to sex and gender identity that can impact the microbiota, extending beyond the influence of sex steroids [49]. It is worth noting that many of these studies did not factor in menopausal status, which signifies a pivotal juncture in a woman's life cycle marked by profound hormonal changes. Therefore, precious evidence is furnished by a study encompassing both pre and post-menopausal women, as well as men [18]. This study demonstrated that the gut microbiota of post-menopausal women exhibited greater similarity to that of men rather than pre-menopausal women [18]. Comprehensive analyses of metagenome functionality unveiled no significant disparities between post-menopausal women and men [18]. These findings underscore the impact of sex-specific hormonal status in shaping sex-specific patterns within the gut microbiota. Nonetheless, due to the cross-sectional design and the absence of a longitudinal within-subject approach with a high degree of covariate stability, this study cannot definitively assert that the observed changes are exclusively linked to sex steroid status. Accumulating evidence suggests that the gut microbiota may play a significant role in determining the timing of puberty through its metabolic and hormonal effects [54]. However, data on the gut microbiota of human adolescents are limited. Korpela et al. demonstrated that the gut microbiota shifts towards an adult-like composition as puberty progresses, indicating a potential association between sex hormones and gut microbiota development. Unfortunately, the lack of hormone level measurements and reliance on 16S rRNA gene sequencing in this study limits direct comparability with our findings [55].

Our results suggest that GAHT induces specific transitional changes in bacterial species that align with the desired gender features. This notably pertains to the species *Parabacteroides goldsteinii*, *Coprococcus sp. ART55/1*, *Coprococcus eutactus*, and *Escherichia coli*. *Coprococcus* species inhabit the human colon and are strictly anaerobic bacteria [56]. More pronounced alterations in the gut metagenome became evident in response to GAHT when we analyzed metagenomic functionality. This analysis revealed significant variations in both the overall composition and a multitude of individual metabolic pathways. Remarkably, among the pathways exhibiting a stronger association with the male hormonal phenotype, several functions related to lipid metabolism were identified. These included processes such as palmitate biosynthesis, unsaturated fatty acids biosynthesis (predominantly by *E. coli*), and fatty acid and beta oxidation IV. While considerable focus has been directed towards investigating the short-chain fatty acid metabolism of the gut microbiota, substantially less research has been undertaken to elucidate the importance of its anabolic and catabolic metabolism of medium- and long-chain fatty acids [57]. The majority of research examining the interplay between dietary lipids and the microbiota is derived from rodent studies. However, these investigations are inherently constrained by the fundamental disparity in nutritional requirements between humans and mice [57].

Transgender and gender-diverse patients encounter numerous barriers to medical care, with a 2019 systematic review indicating that 27% (range, 19–40%) had been denied care by a healthcare professional [9]. Despite ongoing barriers, the knowledge base on transgender healthcare has expanded in recent years and is a rapidly advancing interdisciplinary field [14, 58, 59], The World Professional Association for Transgender Health (WPATH) recognizes that GAHT, while offering benefits such as reduced depression and anxiety, is also associated with gender-specific risks that require consideration during and after transition [58]. There is a well-recognized sexual dimorphism in the risk of various common diseases. Notably, men and postmenopausal women exhibit an elevated cardiometabolic risk in contrast to premenopausal women [60]. A recent meta-analysis revealed that transgender individuals exhibit a 40% increased risk of cardiovascular disease in comparison to cisgender individuals of congruent birth sex [61]. Individuals assigned female at birth and presently identifying as transgender exhibited 2.66 times higher odds of cardiovascular disease in comparison to those identifying as cisgender [62]. The importance of both sex steroids and gut microbiota in cardiometabolic health is well established [63]. Various bacterial metabolites within the intestinal tract, encompassing toll-like receptor agonists, short-chain fatty acids (SCFAs), and secondary bile acids, exert direct influence on metabolic functions such as insulin sensitivity, alongside immune-mediated metabolic impacts [64]. Human studies have revealed the pivotal role of gut microbiota composition and microbial metabolites in predicting major adverse cardiovascular events (MACE) as well as the metabolic and inflammatory alterations preceding MACE [65, 66]. In our study, we found a gender congruent decline of *C. eutactus* in trans men. *C. eutactus*, as a significant producer of butyrate, exhibits firmly established anti-inflammatory properties [67, 68]. Its protective role in glucose homeostasis and insulin sensitivity [69, 70], its association with longevity in centenarians [71], and its potential role in enhancing exercise capacity [72] suggest that it could serve as a pivotal beneficial commensal for promoting cardiometabolic health. Therefore, it can be hypothesized that the decline of *C. eutactus* and potentially other microbiota alterations could be associated with an increased cardiovascular risk in transgender men. These taxonomic findings are supported by multiple alterations in microbiota lipid metabolism during GAHT in our cohort, providing further insights into the implications of microbiota-lipid interactions on body composition, insulin sensitivity, inflammation, and cardiovascular disease risk. Moreover, *C. eutactus* is acknowledged for its neuroprotective potential [68]. Notably, *C. eutactus* has consistently been found to be reduced in individuals with Parkinson's disease (PD), a neurodegenerative movement disorder that affects men more frequently than women [68]. Estrogen is traditionally regarded as a protective factor against PD. Estrogen therapy in postmenopausal cisgender women is associated with a reduced risk of developing PD, and low-dose oral estrogen therapy in postmenopausal cisgender women with PD has demonstrated efficacy in alleviating motor symptoms [73]. Deeb and colleagues suggest that GAHT may influence both motor and non-motor symptomatology in movement disorders, advocating for the expansion of sexual and gender minority research in this field. Alterations in the abundance of *C. eutactus* during GAHT may offer a connection between hormonal levels and PD. Another well-recognized concern in transgender healthcare is the potential for GAHT to increase the risk of hormone-sensitive cancers. Testosterone may promote the formation of colorectal adenomas through unknown mechanisms, potentially explaining the observed higher susceptibility to colorectal cancer (CRC) in men [74]. Among the various factors that may contribute to CRC, *E. coli* strains, particularly those producing colibactin from their polyketide synthesis (pks) locus, have now been identified as potential contributors [75]. We observed a gender-concordant increase of *E. coli* in transgender men. This could potentially elevate their future risk of developing CRC. Currently, no studies have investigated the CRC rate in transgender men compared to cisgender men. However, it is known that transgender individuals utilize the recommended CRC screening less frequently than cisgender individuals [76], which is concerning. Driven by rapid technological advancements, there is a burgeoning interest in leveraging the gut microbiota as both an early indicator of impending disease and a modifiable risk factor amenable to finely tuned therapeutic interventions targeting the microbiota, such as prebiotics, bacteriophages, bacterial metabolites, and engineered probiotics [77]. Our results demonstrate gender-congruent alterations in specific bacterial species that are strongly associated with diseases exhibiting clear gender predisposition, supported by multiple lines of evidence. Since the advent of the NGS revolution, a significant portion of studies has focused either on descriptive analyses in humans or, when intervention was pursued, on animal models. However, in recent years, randomized controlled trials (RCTs) in humans have emerged, providing evidence that microbiota-targeted therapeutics hold great potential for translation into clinical practice. Specifically, with regard to *C. eutactus*, an RCT utilizing galacto-oligosaccharides (GOS) to expand *C. eutactus* among other butyrate producers has demonstrated a reduction in symptoms among patients with lactose intolerance [78]. This illustrates how, in the future, gut microbiota biomarkers could be utilized for post-transition health monitoring or as targets to mitigate modifiable disease risks associated with GAHT through precision microbiota therapeutics.

There is a longstanding hypothesis that the intestinal microbiota influences systemic estrogen levels [79–83]. Microbial–mammalian symbioses frequently exist within complex, co-evolved functional systems that provide mutual benefits. In the intestines, symbiotic bacteria express β-glucuronidase enzymes, which can remove the inactivating glucuronic acid from glucuronidated compounds to use as a carbon source [84]. These bacteria can either further metabolize the parent compound or release it into the gastrointestinal lumen. Compounds that were previously glucuronidated and subsequently reactivated by gastrointestinal microbial β-glucuronidase enzymes can be reabsorbed into the plasma and undergo cycles of enterohepatic recirculation [85]. β-glucuronidase activity influences levels of non-ovarian estrogens via this enterohepatic circulation, supporting the hypothesis that decreased β-glucuronidase levels might increase systemic estrogen levels [86]. Conversely, intestinal β-glucuronidase activity can respond to alterations in female sex hormone status, as evidenced by studies using murine ovariectomy models [87]. Interestingly, our study found that an increase in *E. coli*, a major source of intestinal β-glucuronidase [88], was strongly associated with androgenization and decreased with feminization, suggesting a potential role in hormonal phenotype transition.

The significance of our study transcends the specific population of transgender individuals undergoing GAHT. The findings provide insights into how hormonal treatments, in general, can influence gut microbiota. This has implications for various medical conditions and treatments that involve hormone therapy, such as menopause, andropause, and hormone replacement therapy in cisgender individuals [89, 90]. The gut microbiota is known to play a pivotal role in systemic health, influencing immune function, metabolism, and even mental health [91]. By elucidating the specific changes in gut microbiota induced by GAHT, our study contributes to a broader understanding of how hormonal fluctuations can affect these critical aspects of health. Our research supports the move towards personalized medicine by highlighting the need for tailored healthcare approaches based on individual hormonal profiles and microbiota compositions.

What remains unclear in human trials designed like ours are the mechanisms by which hormonal alterations might influence gut microbiota composition and function. One intriguing hypothesis is that hormonal alterations affect gut transit time. Accumulating evidence suggests that gut transit time is a key factor in shaping the composition and activity of the gut microbiota [92]. Human reproductive hormones influence gastrointestinal transit time. In a seminal study conducted by Wald et al., it was demonstrated that during the luteal phase, characterized by heightened progesterone levels, there was a notable prolongation of gastrointestinal transit time compared to the follicular phase [93]. Notably, a reciprocal relationship between gut transit time and sex steroids appears evident, as elucidated in a human experimental trial wherein pharmacologically induced deceleration of transit time led to a reduction in estrogen levels [94]. Among the differentially abundant taxa observed in our study were *Coprococcus spp.*, which are recognized as indicative of prolonged gut transit time [92]. Future investigations concerning the interplay between human sex hormones and the microbiota should incorporate assessments of gut transit time.

Emerging evidence indicates that bidirectional gut–brain signaling constitutes a communication pathway that utilizes neural, hormonal, and immunological mechanisms to regulate homeostatic processes [95]. Currently, this bidirectional communication is somewhat overlooked, as most research emphasizes the pathway from the microbiota and gut to the brain, such as the role of gut-derived inflammatory signals in the development of psychopathology [96]. Based on recent high-quality evidence from both clinical data and murine models, it has been demonstrated that psychological factors, notably psychological stress, can significantly impact gut homeostasis [97, 98]. Therefore, it is a compelling hypothesis to investigate how the substantial psychological experiences associated with the initial transition period might contribute to the reshaping of gut and microbiota homeostasis.

Our study has limitations that warrant consideration. First, the sample size was relatively small and heterogenous with regard to delivery of GAHT (e.g., oral vs. transdermal estradiol), which restricted our ability to comprehensively explore potential covariates. Second, recognizing the potential advantage of incorporating similarly processed cisgender controls to establish a baseline for sex-specific microbiota patterns, our study’s exclusive reliance on participants before GAHT potentially restricts the generalizability of our findings. Third, since our study did not include a control group receiving placebo instead of GAHT, we are unable to definitively prove that the observed changes in gut microbiota within our cohort occurred exclusively due to alterations in the sex hormone phenotype. However, due to ethical considerations, implementing such a study design will remain untenable in the future as well. Future research should also aim to expand the functional analysis by incorporating both bacterial and host metabolomes. This study focused on trans women and trans men. Contrary to the common assumption that non-binary individuals do not pursue GAHT, recent evidence, such as an Australian survey, indicates a 77.3% demand for GAHT among non-binary individuals [99]. Future research should also examine the implications of GAHT on the gut microbiota in non-binary individuals.

## Conclusions

In conclusion, our findings demonstrate significant differences in the taxonomic and functional profiles of the gut microbiota based on sex hormone profiles. We propose that GAHT leads to the androgenization of the microbiota in trans men and the feminization of the microbiota in trans women, potentially bearing consequences for physiological and health-related outcomes.

## Supplementary Information

Additional file 1. Dietary questionnaire in both German and English translations used in the study.

Additional file 2. Kit instructions for the stool collection tube.

Additional file 3: Fig. S1. Hormonal changes in response to GAHT. The serum hormone levels (in nmol/L) before and 12 weeks after the initiation of GAHT in trans women and trans men are displayed, including (A) Testosterone, (B) Androstenedione, (C) Dehydroepiandrosterone, (D) Dihydrotestosterone, (E) 17-Hydroxyprogesterone, and (F) Estradiol.

Additional file 4. Tabular overview of the GAMM results for all 500 microbial metabolic pathways identified by HUMAnN 3.0 in the HMP Unified Metabolic Analysis Network, including estimates, standard errors, *p*-values, and FDR-corrected *p*-values for the interaction between transition time point and gender.

## Data Availability

The original NGS data and meta data can be obtained by making a reasonable request to the corresponding author. The code to reproduce the results is publicly available on GitHub (https://github.com/Gavaguy/Transgender).

## Abbreviations

ANOVA: Analysis of variance
AUROC: Area under the receiver operating characteristic curve
BL: Baseline
BMI: Body mass index
CRC: Colorectal cancer
DNA: Deoxyribonucleic acid
DGGE: Denaturing gradient gel electrophoresis
FDR: False discovery rate
GAHT: Gender-affirming hormonal treatment
GAMM: Generalized additive mixed model
GO: Gene Ontology
GOS: Galacto-oligosaccharides
HIV: Human immunodeficiency viruses
HRT: Hormone replacement therapy
ICD-10: International Classification of Diseases, 10th Edition
KEGG: Kyoto Encyclopedia of Genes and Genomes
MACE: Major adverse cardiovascular events
PCR: Polymerase chain reaction
PD: Parkinson’s disease
PERMANOVA: Permutational multivariate analyses of variance
RCT: Randomized controlled trial
rRNA: Ribosomal ribonucleic acid
SCFAs: Short chain fatty acids
SE: Standard error
STROBE: Strengthening the Reporting of Observational Studies in Epidemiology
WPATH: World Professional Association for Transgender Health
