# Correction: Gender-affirming hormonal therapy induces a gender-concordant fecal metagenome transition in transgender individuals

**DOI:** 10.1186/s12916-025-04144-5

**Published:** 2025-05-27

**Authors:** Timur Liwinski, Matthias K. Auer, Johanna Schröder, Ina Pieknik, Christian Casar, Dorothee Schwinge, Lara Henze, Günter K. Stalla, Undine E. Lang, Alina von Klitzing, Peer Briken, Thomas Hildebrandt, Jeanne C. Desbuleux, Sarah V. Biedermann, Paul-Martin Holterhus, Corinna Bang, Christoph Schramm, Johannes Fuss

**Affiliations:** 1 Clinic for Adult Psychiatry, University Psychiatric Clinics, University of Basel, Wilhelm Klein-Strasse 27, CH-4002 Basel, Switzerland; 2 Medizinische Klinik and Poliklinik IV, Klinikum Der Universität München, LMU München, Munich, Germany; 3 Institute of Forensic Psychiatry and Sex Research, Center for Translational Neuro- and Behavioral Sciences, University of Duisburg-Essen, Alfredstr. 68-72, 45130 Essen, Germany; 4 Department of Psychology, Institute for Clinical Psychology and Psychotherapy, Medical School Hamburg, Hamburg, Germany; 5 First Department of Medicine, University Medical Centre Hamburg-Eppendorf (UKE), Hamburg, Germany; 6 Medicover Neuroendocrinology, Munich, Germany; 7 Institute for Sex Research, Sexual Medicine and Forensic Psychiatry, University Medical Center Hamburg-Eppendorf, Hamburg, Germany; 8 Department of Gynecology and Obstetrics, CCC Erlangen EMN, Friedrich Alexander University, Erlangen, Germany; 9 Department of Psychiatry and Psychotherapy, Social and Emotional Neuroscience Group, Center for Psychosocial Medicine, University Medical Center Hamburg-Eppendorf, Hamburg, Germany; 10 Division of Pediatric Endocrinology and Diabetes, Department of Children and Adolescent Medicine I, University Hospital of Schleswig-Holstein, Campus Kiel/Christian-Albrechts University of Kiel, 24105 Kiel, Germany; 11 Institute of Clinical Molecular Biology, Christian-Albrechts-University Kiel, University Hospital Schleswig-Holstein, Rosalind-Franklin-Str. 12, 24105 Kiel, Germany; 12 Hamburg Centre for Translational Immunology (HCTI), University Medical Centre Hamburg-Eppendorf, Hamburg, Germany; 13 Martin Zeitz Center for Rare Diseases, University Medical Center Hamburg-Eppendorf, Hamburg, Germany

**Correction: BMC Med 22, 346 (2024)**

**https://doi.org/10.1186/s12916-024-03548-z**

After publication of this article [1], it came to the attention of the authors that the public accession number to the original next-generation sequencing dataset was not provided. The authors have updated the article to include the following information:

The next-generation sequencing data set associated with this study is available in the European Nucleotide Archive (ENA) under the accession number ERP169623, project PRJEB86263. The data can be accessed at https://www.ebi.ac.uk/ena/browser/view/PRJEB86263.
