# Supplementary material for: Gender-affirming hormonal therapy induces a gender-concordant fecal metagenome transition in transgender individuals
Source: BMC Med. 2024 Sep 2;22:346. doi: 10.1186/s12916-024-03548-z (PMC11367877; doi:10.1186/s12916-024-03548-z)
Supplement: Supplementary file 1 — Additional file 1. Dietary questionnaire in both German and English translations used in the study. [file 12916_2024_3548_MOESM1_ESM.pdf]

ERNÄHRUNGS-FRAGEBOGEN

Wie oft verzehren Sie folgende Lebensmittel und Getränke?

	mehrmals täglich	täglich	mehrmals in der Woche	etwa einmal in der Woche	mehrmals im Monat	etwa einmal im Monat	nie	Bemerkungen
Milch, Buttermilch	☐	☐	☐	☐	☐	☐	☐	
Quark, Joghurt, Dickmilch	☐	☐	☐	☐	☐	☐	☐	
Sahne, Crème fraîche	☐	☐	☐	☐	☐	☐	☐	
Käse	☐	☐	☐	☐	☐	☐	☐	
Eier	☐	☐	☐	☐	☐	☐	☐	
Fleisch (ohne Wurstwaren)	☐	☐	☐	☐	☐	☐	☐	
Wurstwaren, Schinken	☐	☐	☐	☐	☐	☐	☐	
Geflügel, Wild	☐	☐	☐	☐	☐	☐	☐	
Fisch	☐	☐	☐	☐	☐	☐	☐	
Gemüse, gegart	☐	☐	☐	☐	☐	☐	☐	
Hülsenfrüchte	☐	☐	☐	☐	☐	☐	☐	
(z.B. Bohnen, Linsen, Erbsen)	☐	☐	☐	☐	☐	☐	☐	
Salate, Rohkost	☐	☐	☐	☐	☐	☐	☐	
Kräuter, frisch	☐	☐	☐	☐	☐	☐	☐	
Obst, frisch	☐	☐	☐	☐	☐	☐	☐	
Kartoffeln	☐	☐	☐	☐	☐	☐	☐	
Teigwaren hell (Nudeln, Spätzle)	☐	☐	☐	☐	☐	☐	☐	
Reis (weiß)	☐	☐	☐	☐	☐	☐	☐	
Vollkornreis, Vollkornnudeln	☐	☐	☐	☐	☐	☐	☐	
Knödel (Kartoffel-, Semmelknödel)	☐	☐	☐	☐	☐	☐	☐	
Pommes frites, Bratkartoffeln	☐	☐	☐	☐	☐	☐	☐	
Mehlspeisen als Hauptgericht	☐	☐	☐	☐	☐	☐	☐	
Haferflocken, Müsli, Cornflakes	☐	☐	☐	☐	☐	☐	☐	
Weißbrot, -brötchen, Toastbrot	☐	☐	☐	☐	☐	☐	☐	
Mischbrot	☐	☐	☐	☐	☐	☐	☐	
Vollkornbrot, -brötchen	☐	☐	☐	☐	☐	☐	☐	
Knäckebrot	☐	☐	☐	☐	☐	☐	☐	
Kuchen, Torten, Gebäck	☐	☐	☐	☐	☐	☐	☐	
Honig, Konfitüre, Nougatcreme	☐	☐	☐	☐	☐	☐	☐	
Schokolade, Pralinen	☐	☐	☐	☐	☐	☐	☐	
sonstige Süßwaren	☐	☐	☐	☐	☐	☐	☐	
(z.B. Bonbons)	☐	☐	☐	☐	☐	☐	☐	
salzige Knabbereien	☐	☐	☐	☐	☐	☐	☐	
(z.B. Chips, Erdnüsse)	☐	☐	☐	☐	☐	☐	☐	
Obst- und Gemüsesäfte	☐	☐	☐	☐	☐	☐	☐	
Mineralwasser, Leitungswasser	☐	☐	☐	☐	☐	☐	☐	
Kräuter-, Früchtetee	☐	☐	☐	☐	☐	☐	☐	
Kaffee, schwarzer Tee	☐	☐	☐	☐	☐	☐	☐	
Limonade, Colagetränke	☐	☐	☐	☐	☐	☐	☐	
Bier	☐	☐	☐	☐	☐	☐	☐	
Wein	☐	☐	☐	☐	☐	☐	☐	
Spirituosen	☐	☐	☐	☐	☐	☐	☐	
Diätgetränke	☐	☐	☐	☐	☐	☐	☐	

How often do you consume the following items?

	Several times a day	Every day	Several times a week	Several times a moth	About once a months	Never	Comment
Milk, buttermilk							
Quark (curd), yogurt, sour milk							
Cream, crème fraîche							
Cheese							
Eggs							
Meat (without sausages)							
Sausages, ham							
Poultry, game							
Fish							
Vegetables, cooked							
Legumes							
(e.g. beans, lentils, peas)							
Salads, raw vegetables							
Fresh herbs							
Fresh fruit							
Potatoes							
White pasta (noodles, spaetzle)							
White rice							
Whole grain rice, whole grain pasta							
Dumplings (potato, bread dumplings)							
French fries, fried potatoes							
Pastries as main dishes							
Oatmeal, muesli, cornflakes							
White bread, rolls, toast							
Mixed bread							
Whole grain bread, rolls							
Crispbread							
Cakes, tarts, pastries							
Honey, jam, nougat cream							
Chocolate, pralines							

How often do you consume the following items?

Other sweets							
(e.g. candies)							
Salty snacks							
(e.g. chips, peanuts)							
Fruit and vegetable juices							
Mineral water, tap water							
Herbal, fruit tea							
Coffee, black tea							
Lemonade, cola drinks							
Beer							
Wine							
Spirits							
Diet drinks							
