# Supplementary material for: Gender-affirming hormonal therapy induces a gender-concordant fecal metagenome transition in transgender individuals
Source: BMC Med. 2024 Sep 2;22:346. doi: 10.1186/s12916-024-03548-z (PMC11367877; doi:10.1186/s12916-024-03548-z)
Supplement: Supplementary file 2 — Additional file 2. Kit instructions for the stool collection tube. [file 12916_2024_3548_MOESM2_ESM.pdf]

INVITEK Molecular

Stool Collection Tube with DNA Stabilizer

1.
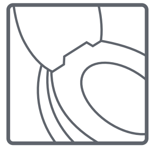

2.
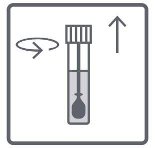

3.
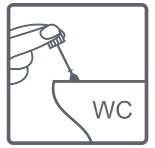

4.
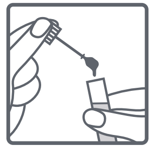

5.
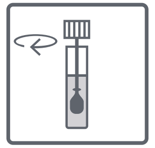

6.
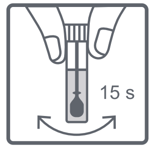

7.
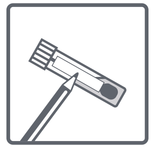

Versionsnummer: 1E7f15/02/2020

DEUTSCH

Stool Collection Tube with DNA Stabilizer
Gebrauchsanweisung

Bitte lesen Sie diese Gebrauchsanweisung sorgfältig durch, bevor Sie das Produkt verwenden.

VERWENDUNGSZWECK:
Das Produkt dient zur Sammlung von Stuhlproben für die DNA-Stabilisierung, -Lagerung und –Extraktion (PSP® Spin Stool DNA Plus Kit / PSP® Spin Stool DNA Basic Kit). Die DNA ist in der DNA-Stabilisierungslösung für drei Monate bei Raumtemperatur stabil.

Das Produkt ist unter Aufsicht von Sachkundigen, wie z.B. technischen Angestellten oder Ärzten, die in der Anwendung molekularbiologischer Methoden geschult sind, zu verwenden.

INHALT:
Stool Collection Tube mit Löffel im Deckel, Etikett zur Probenbeschriftung. Das Röhrchen enthält ungefähr 8 ml DNA-Stabilisierungsflüssigkeit.

EINSCHRÄNKUNGEN DES VERFAHRENS:
Leeren Sie Ihre Blase bevor Sie mit der Probensammlung beginnen. Die Probengröße sollte NICHT die Größe des Löffels überschreiten. Die optimale Probengröße entspricht die einer kleinen Bohne. Falls die Stuhlprobe flüssig sein sollte, dann geben Sie 4 – 5 Löffel von der Probe in das Röhrchen.

WICHTIG:
Vor Gebrauch sollte das Stool Collection Tube Raumtemperatur haben.

1. Sammeln Sie die Stuhlprobe ohne Urin oder Toilettenwasser. Toilettenpapier oder Tücher können erforderlich sein.

2. Schrauben Sie den Deckel von dem Röhrchen ab. Lagern Sie das Röhrchen aufrecht. Verschütten Sie NICHT die Stabilisierungsflüssigkeit.

3. Benutzen Sie den integrierten Löffel im Deckel, um eine kleine Stuhlprobe zu nehmen. Sammeln Sie NICHT zu viel Probe.

4. Geben Sie den Löffel mit der Probe vorsichtig in das Röhrchen mit der Stabilisierungsflüssigkeit.

5. Schrauben Sie den Deckel mit dem Löffel wieder fest auf das Röhrchen.

6. Schütteln Sie das verschlossene Röhrchen für 15 Sekunden, um die Stuhlprobe mit der Stabilisierungsflüssigkeit zu mischen.

7. Beschriften Sie das Röhrchen mit Name, Geburtstag und Datum.

LAGERUNG:
Bei Raumtemperatur (15–30 °C) lagern.

VERSAND:
Versenden Sie die Stuhlprobe entsprechend der Angaben des Anbieters des Stool Collection Tubes. Versenden Sie entsprechend der lokal zutreffenden Richtlinien und Regularien wie UN 3373 (biologische Substanz, Kategorie B).

SICHERHEITSMASSNAHMEN:
Benutzen Sie das Stool Collection Tube NICHT, wenn der Inhalt des Röhrchens verschüttet ist.
Entfernen Sie NICHT die DNA-Stabilisierungsflüssigkeit aus dem Röhrchen.
Inhalt des Röhrchens NICHT einnehmen.
Von Kindern fernhalten. Falls die Stabilisierungsflüssigkeit mit Haut oder Augen in Berührung kommt, spülen Sie die betroffene Stelle mit Wasser. Das Röhrchen vor Erwärmung und direktem Sonnenlicht schützen. Flüssigkeit nur im Originalröhrchen aufbewahren.

ETIKETTENSYMBOLE:
- Packungsbeilage beachten
- Bei Raumtemperatur (15 – 30 °C) lagern.
- Nicht wiederverwenden
- Verfallsdatum
- LOT Lotnummer
- Invitek Molecular GmbH
Robert-Rössle-Str. 10
13125 Berlin, Deutschland

+49 30 9489 2901
info@invitek-molecular.com
www.invitek-molecular.com

ENGLISH

Stool Collection Tube with DNA Stabilizer
Instructions

Please read these instructions carefully before using the product.

INTENDED USE:
Collection of human stool samples for DNA stabilization, storage and extraction (PSP® Spin Stool DNA Plus Kit / PSP® Spin Stool DNA Basic Kit). The DNA is protected from degradation for up to 3 month at room temperature in the DNA Stabilizer.

The product is intended for use under supervision of professionals only, such as technicians, physicians and biologist trained in molecular biological techniques.

CONTENTS:
Stool Collection Tube with integrated spoon in lid, label for donor description, each tube contains 8 ml of DNA Stabilizer reagent.

LIMITATIONS OF THE PROCEDURE:
Empty your bladder before beginning the collection. The sample size should NOT exceed the size of the spoon. Optimal is a sample size comparable to a small bean. When collecting liquid fecal sample transfer about 4 - 5 spoons of sample into the Stool Collection Tube.

IMPORTANT:
Before use make sure that the Stool Collection Tube has room temperature.

1. Collect fecal sample free of urine or toilet water. Toilet paper or tissues may be required.

2. Unscrew the lid of the Stool Collection Tube.
Store the tube upright. Do NOT spill the stabilizing liquid in the tube.

3. Use the integrated spoon in the lid to collect a small stool sample. Do NOT collect too much sample.

4. Carefully transfer the spoon with the sample into the Stool Collection Tube with DNA Stabilizer.

5. Screw lid tightly onto the tube again.

6. Shake tube for 15 sec to mix fecal sample with DNA Stabilizer reagent.

7. Label the tube with donor name, birth date and collection date using provided label.

STORAGE:
Store at room temperature 15 – 30°C (59 - 86°F).

SHIPMENT:
Ship collected stool samples according to the instructions of Stool Collection Tube provider. Ship according to locally applicable guidelines and regulations such as UN 3373 (Biological Substance, Category B).

SAFETY PRECAUTIONS:
Do NOT use the Stool Collection Tube if liquid content of the tube is spilled.
Do NOT remove DNA Stabilizer reagent from the tube.
Do NOT ingest the contents of the tube.
Keep out of the reach of children. Wash with water if DNA Stabilizer reagent comes in contact with skin or eyes. Protect container from warming up and direct sunlight. Keep only in the original container.

LABEL:
- Consult operating instructions
- Store at room temperature 15 – 30°C
- Do not reuse
- Expiry date
- LOT Lot number
- Invitek Molecular GmbH
Robert-Rössle-Str. 10
13125 Berlin, Deutschland

+49 30 9489 2901
info@invitek-molecular.com
www.invitek-molecular.com

ESPANOL

Stool Collection Tube with DNA Stabilizer
Instrucciones

Lea detenidamente estas instrucciones antes de usar el producto.

USO PREVISTO:
Recogida de muestras de heces humanas para la estabilización, conservación y extracción de ADN (PSP® Spin Stool DNA Plus Kit / PSP® Spin Stool DNA Basic Kit). En el estabilizador de ADN, el ADN está protegido frente a la degradación durante un máximo de 3 meses a temperatura ambiente.

El producto está concebido para ser utilizado únicamente bajo la supervisión de profesionales, tales como técnicos, médicos y biólogos con formación en técnicas de biología molecular.

CONTENIDO:
Tubo de recogida de heces con cucharilla incorporada en la tapa y etiqueta para descripción del donante; cada tubo contiene 8 ml de reactivo estabilizador de ADN.

LIMITACIONES DEL PROCEDIMIENTO:
Orine antes de recoger la muestra. El tamaño de la muestra NO debe superar el de la cucharilla. El tamaño óptimo de la muestra sería similar a una habichuela pequeña. Al recoger muestras de líquido fecal, transfiera unas 4–5 cucharillas de muestra al tubo de recogida de heces.

IMPORTANTE:
Antes de usar el tubo de recogida de heces, asegúrese de que esté a temperatura ambiente.

1. Recoja muestras fecales sin orina ni agua del inodoro. Puede ser necesario usar papel higiénico o pañuelos de papel.

2. Desenrosque la tapa del tubo de recogida de heces.
Guarde el tubo en posición vertical. NO vierta el líquido estabilizador del tubo.

3. Use la cucharilla incorporada en la tapa para recoger una pequeña muestra de heces. NO recoja una muestra demasiado grande.

4. Transfiera cuidadosamente la cucharilla con la muestra al tubo de recogida de heces con estabilizador de ADN.

5. Vuelva a enroscar la tapa del tubo de modo que quede bien cerrada.

6. Agite el tubo durante 15 segundos para mezclar la muestra fecal con el reactivo estabilizador de ADN.

7. Etiquete el tubo con el nombre y fecha de nacimiento del donante y la fecha de recogida usando la etiqueta proporcionada.

CONSERVACIÓN:
Conservar a temperatura ambiente, entre 15 °C y 30 °C.

ENVÍO:
Envíe las muestras de heces recogidas según las instrucciones del proveedor del tubo de recogida de heces. Realice el envío conforme a las directrices y normativas locales aplicables, como la UN 3373 (Sustancia biológica, categoría B).

PRECAUCIONES DE SEGURDAD:
NO use el tubo de recogida de heces si se vierte el contenido líquido del tubo.
NO retire el reactivo estabilizador de ADN del tubo.
NO ingiera el contenido del tubo.
Manténgalo fuera del alcance de los niños. Lávese con agua en caso de contacto con la piel o con los ojos del reactivo DNA Stabilizer. Proteja el envase del calor y la luz solar directa. Manténgalo únicamente en el envase original.

ETIQUETA:
- Consulte las instrucciones de uso
- Consérvese a temperatura ambiente, a entre 15°C y 30°C
- No reutilizar
- Fecha de caducidad
- LOT Número de lote
- Invitek Molecular GmbH
Robert-Rössle-Str. 10
13125 Berlin, Deutschland

+49 30 9489 2901
info@invitek-molecular.com
www.invitek-molecular.com

FRANÇAIS

Stool Collection Tube with DNA Stabilizer
Instructions

Veuillez lire attentivement les instructions avant d'utiliser le produit.

USAGE PRÉVU:
prélèvement d'échantillons de selles humaines pour la stabilisation, la conservation et l'extraction de l'ADN (PSP® Spin Stool DNA Plus Kit/ PSP ® Spin Stool DNA Basic Kit). L'ADN est protégé contre la dégradation pendant trois mois maximum à température ambiante dans le stabilisateur d'ADN.

Le produit est destiné à être utilisé uniquement sous la supervision de professionnels, tels que les techniciens, les médecins et les biologistes formés aux techniques biologiques moléculaires.

CONTENU:
Tube de prélèvement des selles avec une cuillère intégrée dans le capuchon, étiquette pour la description du donneur ; chaque tube contient 8 ml de réactif de stabilisation d'ADN.

LIMITES DE LA PROCÉDURE:
vider votre vessie avant de commencer le prélèvement. La taille de l'échantillon ne doit PAS dépasser la taille de la cuillère. La taille d'échantillon optimale doit être comparable à celle d'un petit haricot. Lors du prélèvement d'un échantillon fécal liquide, transférer environ 4 à 5 cuillères d'échantillon dans le Tube de prélèvement des selles.

IMPORTANT:
avant toute utilisation, s'assurer que le Tube de prélèvement des selles est à température ambiante.

1. Prélever un échantillon fécal exempt d'urine ou d'eau des toilettes. Du papier toilette ou des mouchoirs en papier peuvent être requis.

2. Dévisser le capuchon du Tube de prélèvement des selles.
Conserver le tube en position verticale. Ne PAS déverser le liquide de stabilisation dans le tube.

3. Utiliser la cuillère intégrée dans le capuchon pour prélever un petit échantillon de selles. Ne PAS prélever une quantité trop importante d'échantillon.

4. Transférer soigneusement la cuillère avec l'échantillon dans le Tube de prélèvement des selles contenant un stabilisateur d'ADN.

5. Revisser fermement le couvercle sur le tube.

6. Agiter le tube pendant 15 secondes pour mélanger l'échantillon fécal avec le réactif de stabilisation de l'ADN.

7. Étiqueter le tube avec le nom du donneur, la date de naissance et la date de prélèvement en utilisant l'étiquette fournie.

CONSERVATION:
conserver à température ambiante entre 15 et 30 °C.

LIVRASION:
livrer les échantillons de selles prélevés conformément aux instructions du fournisseur du Tube de prélèvement des selles. Expédier conformément aux directives et réglementations locales en vigueur telles que UN 3373 (Substance biologique, catégorie B).

MESURES DE SÉCURITÉ:
Ne PAS utiliser le Tube de prélèvement des selles en cas d'écoulement du contenu liquide du tube.
Ne PAS retirer le réactif de stabilisation d'ADN du tube.
Ne PAS ingérer le contenu du tube.
Tenir hors de portée des enfants. Laver à l'eau si le réactif de DNA Stabilizer entre en contact avec la peau ou les yeux. Protéger le contenant contre le réchauffement et la lumière directe du soleil. Conserver uniquement dans le contenant d'origine.

ÉTIQUETTE:
- Consulter le mode d'emploi
- Conserver à température ambiante, entre 15 et 30 °C
- Ne pas réutiliser
- Date de péremption
- LOT Numéro de lot
- Invitek Molecular GmbH
Robert-Rössle-Str. 10
13125 Berlin, Deutschland

+49 30 9489 2901
info@invitek-molecular.com
www.invitek-molecular.com

INVITEK Molecular

Stool Collection Tube with DNA Stabilizer

1.
2.
3.
4.
5.
6.
7.

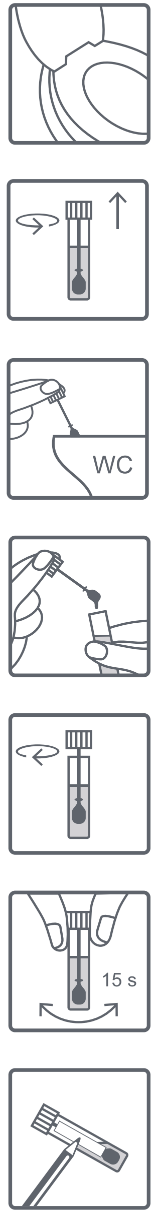

Versionsnummer: 1E7f15/02/2020

ITALIANO

Stool Collection Tube with DNA Stabilizer
Istruzioni

Si prega di leggere attentamente queste istruzioni prima di utilizzare il prodotto.

USO PREVISTO:
Raccolta di campioni di feci umane per la stabilizzazione, conservazione ed estrazione di DNA (PSP® Spin Stool DNA Plus Kit / PSP® Spin Stool DNA Basic Kit). Il DNA è protetto dal deterioramento fino a 3 mesi a temperatura ambiente nel DNA Stabilizer.

Il prodotto deve essere utilizzato unicamente sotto la supervisione di professionisti quali tecnici, medici e biologi con un'adeguata conoscenza delle tecniche biologiche molecolari.

CONTENUTO:
Provetta per la raccolta di feci con cucchiaio integrato nel tappo, etichetta per la descrizione del donatore, ogni provetta contiene 8 ml di DNA Stabilizer.

LIMITI DELLA PROCEDURA:
Svuotare la vescica prima di procedere alla raccolta. La dimensione del campione NON deve superare la dimensione del cucchiaio. Il campione ideale ha una dimensione paragonabile a quella di un piccolo fagiolo. Se si raccoglie un campione di feci liquide, trasferire circa 4-5 cucchiai di campione all'interno della provetta.

IMPORTANTE:
Prima dell'uso, assicurarsi che la provetta per la raccolta delle feci sia a temperatura ambiente.

1. Raccogliere un campione di feci privo di urina o acqua del water. Potrebbe essere necessario utilizzare carta igienica o fazzoletti.

2. Svitare il tappo della provetta per la raccolta delle feci. Conservare la provetta in posizione verticale. NON versare il liquido stabilizzante nella provetta.

3. Utilizzare il cucchiaio integrato nel tappo per raccogliere un piccolo campione di feci. NON raccogliere un campione troppo abbondante.

4. Trasferire delicatamente il cucchiaio con il campione all'interno della provetta con DNA Stabilizer.

5. Riavvitare il tappo saldamente sulla provetta.

6. Agitare la provetta per 15 sec. per miscelare il campione fecale con il reagente DNA Stabilizer.

7. Applicare l'etichetta sulla provetta dello stabilizzatore con il nome del donatore e la data della raccolta usando l'apposita etichetta.

CONSERVAZIONE:
Conservare a temperatura ambiente a 15 - 30 °C.

SPEDIZIONE:
Spedire i campioni di feci raccolti seguendo le istruzioni del fornitore della provetta. Spedire conformemente alle linee guida locali applicabili e alle normative quali UN 3373 (Sostanze biologiche, categoria B).

PRECAUZIONI DI SICUREZZA:
NON utilizzare la provetta se il liquido contenuto al suo interno è fuoriuscito.
NON rimuovere il reagente DNA Stabilizer dalla provetta.
NON ingerire il contenuto della provetta.
Tenere fuori dalla portata dei bambini.
Lavare con acqua se il reagente DNA Stabilizer entrano in contatto con la pelle o gli occhi.
Proteggere il contenitore dal calore e dalla luce diretta del sole. Conservare solo nel contenitore originale.

ETICHETTA:
Consultare le istruzioni di funzionamento
Conservare a temperatura ambiente 15 – 30 °C
Non riutilizzare
Data di scadenza
LOT Numero di lotto

Invitek Molecular GmbH
Robert-Rössle-Str. 10
13125 Berlin, Deutschland

+49 30 9489 2901
info@invitek-molecular.com
www.invitek-molecular.com

NEDERLANDS

Stool Collection Tube with DNA Stabilizer
Instructies

Lees deze instructies aandachtig voordat u het product gebruikt.

BEOOGD GEBRUIK:
Verzameling van monsters van menselijke ontlasting voor DNA-stabilisatie, opslag en extractie (PSP® Spin Stool DNA Plus Kit/ PSP® Spin Stool DNA Basic Kit). Het DNA wordt gedurende maximaal 3 maanden in het DNA-stabilisatiemiddel bij kamertemperatuur beschermd tegen verslechtering.

Het product is enkel bedoeld voor gebruik onder toezicht van professionals, zoals technici, artsen en biologen die opgeleid zijn in de technieken van moleculaire biologie.

INHOUD:
Buisje voor het opvangen van menselijke ontlasting met geïntegreerde lepel in deksel, label voor donorbeschrijving, elk buisje bevat 8 ml DNA-stabilisatiemiddel.

BEPERKINGEN VAN DE PROCEDURE:
Maak uw blaas leeg voordat u met het opvangen begint. Het monster van uw ontlasting mag NIET groter zijn dan de grootte van de lepel. De optimale grootte van het op te vangen monster is vergelijkbaar met een kleine boon. Zorg dat u ongeveer 4 - 5 lepels vloeibare ontlasting in het buisje opvangt.

BELANGRIJK:
Controleer vóór gebruik of het buisje met het DNA-stabilisatiemiddel op kamertemperatuur is.

1. Verzamel ontlasting waarin geen urine of toiletwater aanwezig is. Voor het opvangen kan toiletpapier nodig zijn.

2. Schroef het deksel van het buisje los. Bewaar het buisje rechtop. Mors de stabilisatievloeistof NIET uit het buisje.

3. Gebruik de geïntegreerde lepel in het deksel om een klein monster van uw ontlasting te verzamelen. Verzamel NIET te veel ontlasting.

4. Breng de lepel met de ontlasting voorzichtig over naar het buisje met het DNA-stabilisatiemiddel.

5. Draai het deksel stevig vast op het buisje.

6. Schud het buisje gedurende 15 seconden om de ontlasting te mengen met het DNA-stabilisatiemiddel.

7. Plak het bijgeleverde etiket met daarop de naam en de geboortedatum van de donor en de datum van afname op het stabilisatiebuisje.

BEWAREN:
Bewaren op kamertemperatuur 15 – 30°C.

VERZENDING:
Verzend het buisje volgens de instructies van de leverancier van het buisje. Verzend het buisje volgens de plaatselijke richtlijnen en regels, zoals UN 3373 (biologische substantie, categorie B).

VEILIGHEIDSMAATREGELEN:
Gebruik het buisje NIET als de vloeibare inhoud ervan uit het buisje gemorst is.
Verwijder het DNA-stabilisatiemiddel niet uit het buisje.
Slik de inhoud van het buisje NIET in.
Buiten het bereik van kinderen bewaren. Afwassen met water indien de DNA Stabilizer in contact komt met de huid of de ogen.Bescherm het buisje tegen opwarmen en direct zonlicht. Uitsluitend in het originele buisje bewaren.

LABELS:
Zie gebruiksaanwijzing
Bewaren bij kamertemperatuur, 15 – 30 °C
Niet opnieuw gebruiken
Vervaldatum
LOT Partijnummer

Invitek Molecular GmbH
Robert-Rössle-Str. 10
13125 Berlin, Deutschland

+49 30 9489 2901
info@invitek-molecular.com
www.invitek-molecular.com

PORTUGUÊS

Stool Collection Tube with DNA Stabilizer
Instruções

Por favor, leia atentamente estas instruções antes de utilizar este produto.

UTILIZAÇÃO PREVISTA:
Colheita de amostras de fezes humanas para estabilização, armazenamento e extração de ADN (PSP® Spin Stool DNA Plus Kit / PSP® Spin Stool DNA Basic Kit). O ADN é protegido da degradação até 3 meses, à temperatura ambiente, no Estabilizador de ADN.

O produto destina-se apenas a ser utilizado sob a supervisão de profissionais, tais como técnicos, médicos e biólogos com formação em técnicas de biologia molecular.

CONTEÚDO:
Tubo de colheita de fezes com colher integrada na tampa, rótulo para descrição do dador, cada tubo contém 8 ml de reagente estabilizador de ADN.

LIMITAÇÕES DO PROCEDIMENTO:
Esvazie a sua bexiga antes de iniciar a colheita. O tamanho da amostra NÃO deve exceder o tamanho da colher. O tamanho de amostra é comparável a um pequeno feijão. Ao efetuar colheita de amostra fecal líquida, transfira cerca de 4 - 5 colheres de amostra para o tubo de colheita de fezes.

IMPORTANTE:
Antes do uso, certifique-se de que o tubo de colheita de fezes possui temperatura ambiente.

1. Colha a amostra fecal livre de urina ou água da sanita. Pode ser necessário papel higiénico ou lenços de papel.

2. Desaperte a tampa do tubo de colheita de fezes. Guarde o tubo na vertical. NÃO derrame o líquido estabilizador no tubo.

3. Use a colher integrada na tampa para colher uma pequena amostra de fezes. NÃO colha demasiada quantidade de amostra.

4. Transfira cuidadosamente a colher com a amostra para o tubo de colheita de fezes com estabilizador de ADN.

5. Aperte firmemente a tampa no tubo novamente.

6. Agite o tubo durante 15 segundos para misturar a amostra fecal com o reagente Estabilizador de ADN.

7. Rotule o tubo com o nome do dador, data de nascimento e a data de recolha, utilizando o rótulo para o efeito.

CONSERVAÇÃO:
Conserve à temperatura ambiente entre 15 e 30 °C.

ENVIO:
Envie as amostras de fezes colhidas de acordo com as instruções do fornecedor do tubo de colheita de fezes. Envie de acordo com as diretrizes e regulamentos locais aplicáveis, tais como a UN 3373 (Substância biológica, Categoria B).

PRECAUÇÕES DE SEGURANÇA:
NÃO use o tubo de colheita de fezes se o conteúdo líquido do tubo for derramado.
NÃO remova o reagente estabilizador de ADN do tubo.
NÃO ingira o conteúdo do tubo.
Manter fora do alcance das crianças.
Lavar com água se o reagente DNA Stabilizer entrar em contacto com a pele ou os olhos.
Proteja o recipiente do aquecimento e luz solar direta. Mantenha apenas no recipiente original.

ROTULAGEM:
Consultar o manual de instruções
Conservar à temperatura ambiente entre 15 a 30 °C
Não reutilizar
Data de validade
LOT Número do lote

Invitek Molecular GmbH
Robert-Rössle-Str. 10
13125 Berlin, Deutschland

+49 30 9489 2901
info@invitek-molecular.com
www.invitek-molecular.com
