# Supplementary figures and images for: Gender-affirming hormonal therapy induces a gender-concordant fecal metagenome transition in transgender individuals
Source: BMC Med. 2024 Sep 2;22:346. doi: 10.1186/s12916-024-03548-z (PMC11367877; doi:10.1186/s12916-024-03548-z)

**A**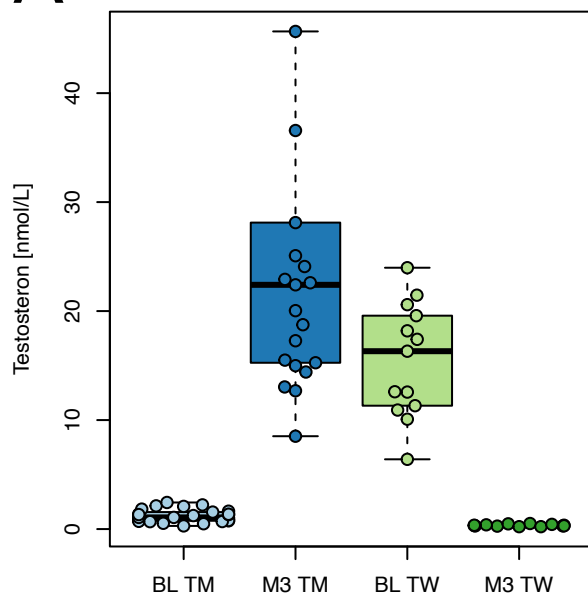**B**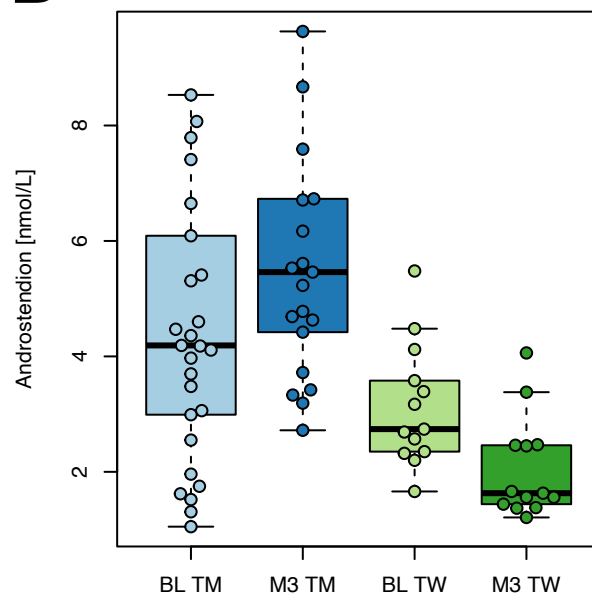**C**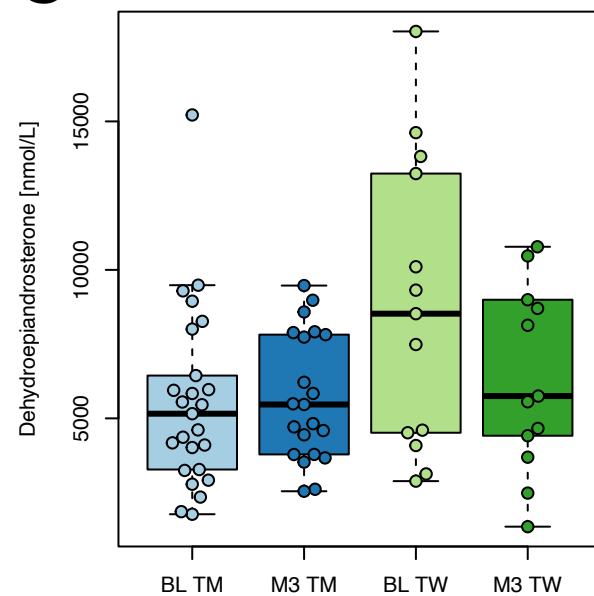**D**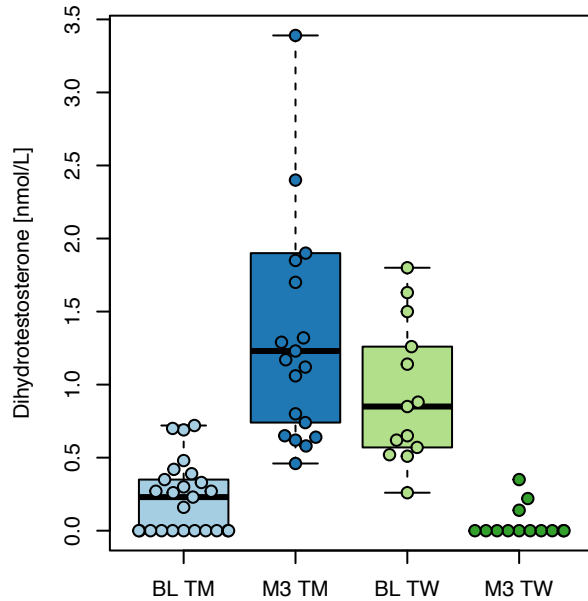**E**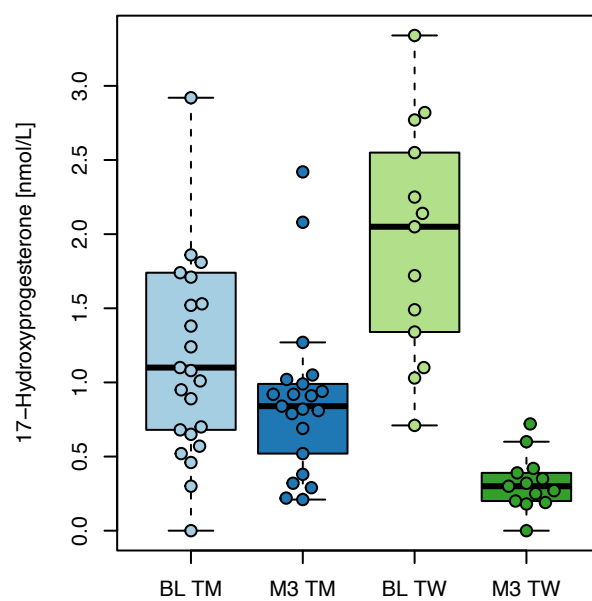**F**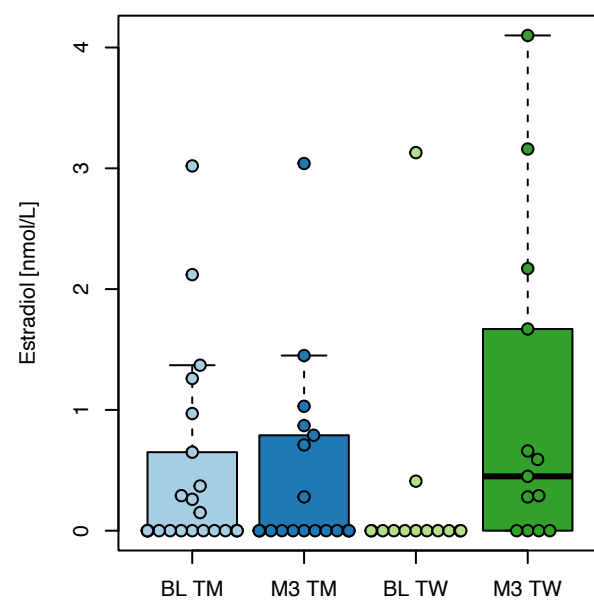

Supplement: Supplementary file 3 — Additional file 3: Fig. S1. Hormonal changes in response to GAHT. The serum hormone levels (in nmol/L) before and 12 weeks after the initiation of GAHT in trans women and trans men are displayed, including (A) Testosterone, (B) Androstenedione, (C) Dehydroepiandrosterone, (D) Dihydrotestosterone, (E) 17-Hydroxyprogesterone, and (F) Estradiol. [file 12916_2024_3548_MOESM3_ESM.pdf]
